# Evaluation of the impact of matrix stiffness on encapsulated HepaRG spheroids

**DOI:** 10.1186/1753-6561-7-S6-P77

**Published:** 2013-12-04

**Authors:** Sofia P Rebelo, Marta Estrada, Rita Costa, Christophe Chesné, Catarina Brito, Paula M Alves

**Affiliations:** 1 iBET, Instituto de Biologia Experimental e Tecnológica, 2780-901 Oeiras, Portugal; 2 Instituto de Tecnologia Química e Biológica, Universidade Nova de Lisboa, 2780-157 Oeiras, Portugal; 3 Biopredic International, Rennes, France (C.C., R.L., S.C

## Background

The drug development process is widely hampered by the lack of human models that recapitulate liver functionality and efficiently predict toxicity of new chemical compounds. Moreover, liver failure is a global medical problem, with transplantation being the only effective treatment currently available. The bipotent liver progenitor cell line HepaRG can be differentiated into cholangiocyte and hepatocyte-like cells that express major functions of mature hepatocytes, representing a valuable tool to model hepatic function [1]. Current two-dimensional (2D) protocols for the differentiation into mature hepatocyte-like cells fail to recapitulate the complex cell-cell interactions, which are crucial for maintaining polarity and inherent mature hepatic functionality. Herein, we present a three-dimensional (3D) strategy for the culture of HepaRG cells based on the encapsulation of aggregates. The effect of matrix stiffness on expansion and differentiation was evaluated through encapsulation with different concentrations of alginate (1.1% and 2%). Further characterization of the hepatic features will reveal the extent of the hepatic functionality of the generated spheroids.

## Materials and methods

HepaRG cells were routinely propagated in static conditions as previously described [2]. Briefly, culture medium Williams E was supplemented with 1% (v/v) Glutamax, 1% (v/v) pen/strep, 5 μ g/ml insulin and 50 μ M hydrocortisone hemissuccinate and 10% (v/v) FBS and cultures were maintained at 37 ° C, 5% CO_2_. Spinner vessels with ball impeller (Wheaton) were inoculated with inoculums ranging from 5 to 8 × 10^5 ^cell/mL and an agitation ranging from 35 to 45 rpm to attain the desired aggregation conditions. Aggregate size was determined by measuring Ferret's diameter using the Image J software (NIH). After 3 days of aggregation, spheroids were encapsulated in 1.1% and 2% (w/v) of Ultra Pure MVG alginate (UP MVG NovaMatrix, Pronova Biomedical) in NaCl 0.9% (w/v) solution. Encapsulation was performed in an electrostatically driven microencapsulation unit VarV1 (Nisco) and cultures were maintained for 14 days in stirred culture conditions. Viability was determined by the double stain viability test - alginate beads were collected from stirred cultures, incubated with fluorescein diacetate (10 μg/mL) and TO-PRO3 ® (1 μM) and observed on a fluorescence microscope (Leica DMI6000) - and by the Trypan blue exclusion method - alginate beads were dissociated with a solution of Sodium citrate 50 mM, Sodium chloride 104 mM and spheroids were dissociated by incubation with Trypsin 0.05%-EDTA (Gibco) and counted trypan blue exclusion dye. For characterization of the cultures, encapsulated spheroids were fixed as previously described [3] and incubated with phalloidin and prolong gold with DAPI and images were acquired in a confocal microscope (Andor spinning disk).

## Results

In 2D cultures, HepaRG cells proliferate until confluence is reached and the cell-cell interactions established associated with the spatial constriction are postulated to trigger the differentiation program and maintain the differentiated state [1,4]. Moreover, the mechanochemical environment has been previously shown to strongly influence the liver-specific functions [5]. Thus, it was hypothesized that the microenvironment created by encapsulation of spheroids with an inert biomaterial with different stiffness levels, would promote differential behavior of the spheroids, towards differentiation or proliferation. Alginate concentrations of 1.1 and 2% (w/v) were used, given the 10 fold difference in stiffness, measured by the elastic modulus [6]. Both viability and the growth profile were monitored throughout culture time.

**Figure 1:**
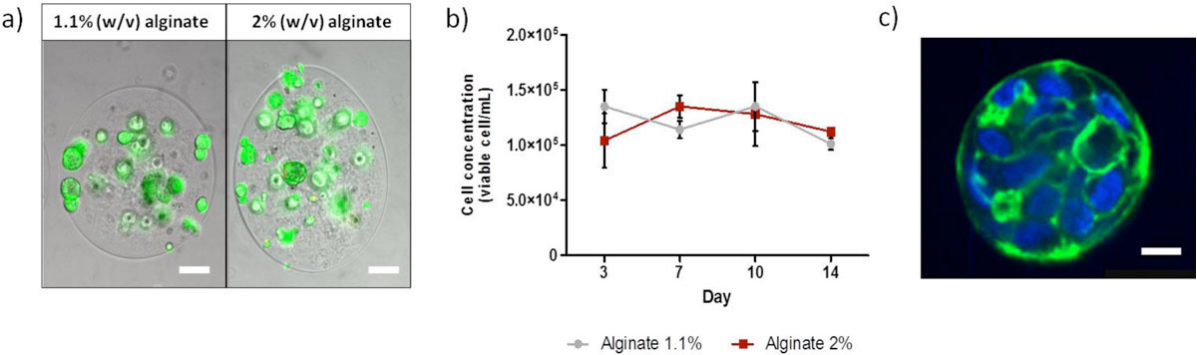
**Characterization of encapsulated cultures of HepaRG spheroids** (A) Viability assessed by staining the encapsulated spheroids with fluorescein diacetate (live, green) and TO-PRO3 ® (dead, red). Spheroids in 1.1 and 2% (w/v) of alginate after 14 days of culture are represented. Scale bar: 100 μm (B) Growth profile of encapsulated cultures of 1.1 and 2% (w/v) of alginate. (C) Immunofluorescence characterization of hepatic spheroids (1.1% alginate) after 14 days of culture. Actin filaments - green; Nuclei - blue. Scale bar: 10 μm.

In both culture conditions, the viability was maintained above 85%, showing that the alginate concentration does not affect diffusion of nutrients or oxygen to supply effectively the cell spheroids (Figure 1 A). Moreover, it was observed that the growth profile was comparable for the two cultures, with growth arrest after aggregation and no proliferation occurring either in both alginate concentrations (Figure 1 B). This suggests that the differentiation program is triggered either in softer and stiffer microenvironments, being 1.1% alginate concentration sufficient to initiate the process.

The structural organization of the cell spheroids in both stiffness environments was characterized by the arrangement of actin filaments, which is associated to the tight junctions in highly polarized epithelial cells. As shown in Figure 1 C, the cells are disposed in a highly polarized manner, without necrotic centres.

## Conclusions

In the current work, the encapsulation of liver spheroids with different stiffness conditions was evaluated as a strategy to culture HepaRG cells. It was observed that the encapsulation with different alginate concentrations is compatible with maintenance of highly viable cultures of liver spheroids, with growth arrest and cell polarization promoted by spatial constriction and the enhanced cell-cell interactions in 3D.
